# Supplementary material for: Effectiveness of PCSK9 inhibitors: A Target Trial Emulation framework based on Real-World Electronic Health Records
Source: PLoS One. 2024 Aug 22;19(8):e0309470. doi: 10.1371/journal.pone.0309470 (PMC11341039; doi:10.1371/journal.pone.0309470)
Supplement: S1 Table — (DOCX) [file pone.0309470.s001.docx]

**Table S1**. List of the ICD9-CM codes used to select the documented ASCVD events

| **Disease** | **ICD9-CM Code** |
| --- | --- |
| Acute Coronary Sindrome (ACS) | 410-Acute myocardial infarction |
| Angina/Chronic Ischemia | 411.x-Other acute and subacute forms of ischemic heart disease 413.x-Angina pectoris 414.x- Other forms of chronic ischemic heart disease |
| Ictus/Transient Ischemic Attack (TIA) | 433.x- Occlusion and stenosis of precerebral arteries 434.x- Occlusion of cerebral arteries 435.x-Transient cerebral ischemia 436.x-Acute, but ill-defined, cerebrovascular disease 437.x-Other and ill-defined cerebrovascular disease |
| Coronary Artery Bypass Graft (CABG)/ Percutaneous transluminal coronary angioplasty (PTCA) | 36.01- Coronary revascularization of single vessel – coronary atherectomy without thrombolytic agent  36.02- Coronary revascularization of single vessel – coronary atherectomy with thrombolytic agent  36.05- Coronary revascularization of multiple vessels – coronary atherectomy with and without thrombolytic agent 36.06-Insertion of non-drug-eluting coronary artery stent(s)  36.1x-Bypass anastomosis for heart revascularization |
| Peripheral Artery Disease (PAD) | 443.9-Peripheral vascular disease, unspecified 433.0x- Occlusion and stenosis of basilar artery 433.1x- Occlusion and stenosis of carotid artery 433.2x- Occlusion and stenosis of vertebral artery 433.3x- Occlusion and stenosis of multiple and bilateral 433.8x- Occlusion and stenosis of other specified precerebral artery 433.9x-Occlusion and stenosis of unspecified precerebral artery 434.x-Occlusion of cerebral arteries 440.2x-Arteriolosclerosis of native arteries of the extremities 442.81-Aneurism of artery of neck 443.81-Peripheral angiopathy in diseases classified elsewhere 444.2x-Embolism of arteries of the extremities 444.81-Embolism of Iliac artery 707.1x-Ulcer of lower limbs, except pressure ulcer  785.4-Gangrene V49.6x-Upper limb amputation status V49.7x-Lower limb amputation status  113-Amputation for circulatory disorders except upper limb and toes  114-Upper limb and toes amputation for circulatory disorders 00.45- Insertion of one vascular stent 00.55-Insertion of drug-eluting stent(s) of other peripheral vessel(s) 00.61-Percutaneous angioplasty of extracranial vessel(s) 00.62-Percutaneous angioplasty of intracranial vessel(s) 00.63-Percutaneous insertion of carotid artery stent(s) 00.64-Percutaneous insertion of other extracranial artery stent(s) 00.65-Percutaneous insertion of intracranial vascular stent(s) 38.02-Incision of vessel, other vessels of head and neck 38.12-Endarterectomy, other vessels of head and neck 38.32-Resection of vessel with anastomosis, other vessels of head and neck 38.42-Resection of vessel with replacement, other vessels of head and neck 38.62-Other excision of vessels, other vessels of head and neck 38.82-Other surgical occlusion of vessels, other vessels of head and neck 39.22-Aorta-subclavian-carotid bypass 39.25-Aorta-iliac-femoral bypass 39.29-Other (peripheral) vascular shunt or bypass 39.50-Angioplasty of other non-coronary vessel(s) 39.90-Insertion of non-drug-eluting peripheral (non-coronary) vessel stent(s) 38.18-Endarterectomy, lower limb arteries 84.0x-Upper limb amputation, not otherwise specified 84.1x-Amputation and disarticulation of finger 84.3x-Amputation through hand  250.7x-Diabetes with peripheral circulatory disorders |

ICD9-CM: International Classification of Diseases, Ninth Revision.
