# Supplementary material for: Effectiveness of PCSK9 inhibitors: A Target Trial Emulation framework based on Real-World Electronic Health Records
Source: PLoS One. 2024 Aug 22;19(8):e0309470. doi: 10.1371/journal.pone.0309470 (PMC11341039; doi:10.1371/journal.pone.0309470)
Supplement: S2 Table — (DOCX) [file pone.0309470.s002.docx]

**Table S2**. List of the ATC codes used to select Lipid Lowering Therapies (LLT) of interest for the study.

| **Substance** | **ATC code** |
| --- | --- |
| Evolocumab | C10AX13 |
| Alirocumab | C10AX14 |
| Atorvastatin (80mg and 40mg) | C10AA05^ |
| Atorvastatin (20mg and 10mg) | C10AA05^ |
| Rosuvastatin (40mg and 20mg) | C10AA07^ |
| Rosuvastatin (10mg and 5mg) | C10AA07^ |
| Lovastatin | C10AA02 |
| Pravastatin | C10AA03 |
| Fluvastatin | C10AA04 |
| Simvastatin | C10AA01 |
| Ezetimibe | C10AX09 |
| Ezetimibe and simvastatin | C10BA02 |
| Ezetimibe and rosuvastatin (10mg and 5mg) | C10BA06^ |
| Ezetimibe and rosuvastatin (40mg and 20mg) | C10BA06^ |
| Ezetimibe and atorvastatin (20mg and 10mg) | C10BA05^ |
| Ezetimibe and rosuvastatin (80mg and 40mg) | C10BA05^ |
| Omega-3 fatty acid | C10AX06 |
| Fibrates | C10AB* |
| Bile acid sequestrants | C10AC* |

^ To distinguish the different dosages, the specific marketing authorization codes were used

ATC: Anatomical Therapeutic Chemical Classification
